# Protective role of vitamin B6 (PLP) against DNA damage in *Drosophila* models of type 2 diabetes

**DOI:** 10.1038/s41598-018-29801-z

**Published:** 2018-07-30

**Authors:** Chiara Merigliano, Elisa Mascolo, Mattia La Torre, Isabella Saggio, Fiammetta Vernì

**Affiliations:** 1 Dipartimento di Biologia e Biotecnologie “Charles Darwin”, Sapienza Università di Roma, Rome, 00185 Italy; 2 Istituto di Biologia e Patologia Molecolari del CNR, Rome, 00185 Italy

## Abstract

Growing evidence shows that improper intake of vitamin B6 increases cancer risk and several studies indicate that diabetic patients have a higher risk of developing tumors. We previously demonstrated that in *Drosophila* the deficiency of Pyridoxal 5′ phosphate (PLP), the active form of vitamin B6, causes chromosome aberrations (CABs), one of cancer prerequisites, and increases hemolymph glucose content. Starting from these data we asked if it was possible to provide a link between the aforementioned studies. Thus, we tested the effect of low PLP levels on DNA integrity in diabetic cells. To this aim we generated two *Drosophila* models of type 2 diabetes, the first by impairing insulin signaling and the second by rearing flies in high sugar diet. We showed that glucose treatment induced CABs in diabetic individuals but not in controls. More interestingly, PLP deficiency caused high frequencies of CABs in both diabetic models demonstrating that hyperglycemia, combined to reduced PLP level, impairs DNA integrity. PLP-depleted diabetic cells accumulated Advanced Glycation End products (AGEs) that largely contribute to CABs as α-lipoic acid, an AGE inhibitor, rescued not only AGEs but also CABs. These data, extrapolated to humans, indicate that low PLP levels, impacting on DNA integrity, may be considered one of the possible links between diabetes and cancer.

## Introduction

Growing evidence associates micronutrients as minerals and vitamins to genome integrity maintenance showing that their deficiency can damage DNA analogously to common carcinogens. Micronutrients act as co-factors or substrates for enzymes that counteract genotoxins as well as enzymes working in DNA metabolism (reviewed in^1^). Vitamins belonging to B group as folates (vitamin B9 and its derivatives) and B12 vitamin act as cofactors of enzymes involved in the conversion of dUMP in dTMP by the transfer of a methyl group. It has been shown that in human cells and rodents^2^ the impairment of this reaction due to folate and B12 deficiency causes Uracil misincorporation, a mutagenic lesion leading to point mutation, micronuclei formation and chromosome aberrations (CABs) that are known to contribute to the genesis and progression of human cancers^3–5^. Another consequence of impaired dUMP conversion is nucleotide pool imbalance that can compromise crucial cellular processes as DNA synthesis and repair. Impairment of DNA repair has been observed in cultured cells and animal models deprived of folates (reviewed in^6^). Thus, it is not surprising that low folate levels have been correlated with some cancers as demonstrated by many epidemiological studies^7–10^.

Pyridoxal-5′-phosphate (PLP), the active form of vitamin B6, plays a role in protecting cells from DNA damage. Mammals produce PLP from vitamers (pyridoxine, pyridoxamine and pyridoxal), which are introduced by the diet and recycled in a “salvage pathway” through the action of two enzymes, Pyridoxal kinase and Pyrodoxine/pyridoxamine oxidase. PLP serves as a co-enzyme in approximately 160 enzyme reactions that regulate metabolism of glucose, lipids, amino acids, heme, DNA/RNA and many neurotransmitters^11–13^. In addition to work as cofactor, vitamin B6 plays antioxidant roles by counteracting the formation of genotoxic compound, the Advanced Glycation End-products (AGEs)^14,15^ and by quenching oxygen reactive species^16,17^. Given its role in numerous metabolic reactions PLP has been associated to many human disorders including diabetes and cancer but underlying mechanisms are not fully elucidated (reviewed in^14,18–20^). High expression level of Pyridoxal kinase (PDXK) has been positively correlated with survival of nonsmall cell lung cancer (NSCLC) patients^21^. In addition, Vitamin B6 intake and blood PLP levels were inversely correlated with the colorectal cancer risk^22^.

We recently demonstrated in *Drosophila* that PLP deficiency caused either by mutations in the pyridoxal kinase-coding gene (*dPdxk*) or by vitamin B6 antagonists results in chromosome aberrations (CABs) suggesting a role for Pyrodoxal kinase in chromosome integrity maintenance. This function is evolutionarily conserved in human and yeast as PDXK depletion by RNA interference in HeLa cells induces CAB formation^23^ and mutations in *BUD16* gene, encoding *Saccharomyces cerevisiae* Pyridoxal kinase, result in gross chromosome rearrangements^24^. More interestingly CAB frequency in *Drosophila* and human PLP-depleted cells was strongly enhanced by glucose treatment. In addition, PLP deficiency increases glucose content in *dPdxk*^1^ mutant hemolymph and causes AGE accumulation in neuroblasts. Treatment of *dPdxk*^1^ mutant cells with α-lipoic acid (ALA), a well-known AGE inhibitor, lowers both AGE formation and CAB frequency, suggesting a possible AGE-CAB cause-effect relationship. These findings indicate that a high intracellular glucose level has a dramatic clastogenic effect if combined with PLP deficiency and have prompted us to investigate if in diabetes low PLP levels can impair DNA integrity. Epidemiological studies clearly indicate that diabetes increases the risk of many types of cancer with mechanisms not yet fully understood. Possible links between these two diseases are hyperinsulinemia, hyperglycemia and fat-induced chronic inflammation. Several lines of evidence indicate that hyperglycemia may lead to cancer through DNA damage (reviewed in^25^). It is well known that high glucose causes DNA damage by increasing the oxygen reactive species production via several mechanisms (namely the polyol pathway, hexosamine pathway, AGE pathway, and protein kinase C)^26,27^. Interestingly, oxidative damage and DNA strand breaks have been found in both type 1 and type 2 diabetic patients^28–30^.

To investigate if in diabetic cells PLP deficiency can impair genome integrity we used *Drosophila* as a model system. For the past decade *Drosophila* turned out to be a promising organism to model diabetes as flies and humans largely share mechanisms underlying the maintenance of balance between stored and circulating glucose (reviewed in^31^). Insulin pathway signaling, a biochemical pathway well conserved in flies (reviewed in^32^), plays a crucial role in glucose homeostasis maintenance as it triggers the uptake of glucose into liver, adipose tissue and muscle and promotes the storage of these nutrients in the form of glycogen. In humans and mice insulin pathway impairment, due to mutations, cause severe insulin resistance syndromes and type 2 diabetes (reviewed in^33^). Also in *Drosophila* malfunctioning of insulin signaling, induced either by mutations or by feeding flies with a sugar rich diet, produce diabetic hallmarks that mimic type 2 diabetes (reviewed in^34^).

In this work, we generated two different diabetic fly models, the first by depleting three well conserved proteins involved in insulin pathway as InR, Chico (IRS) and Akt1 and the second by rearing flies in sugar rich diet^35^. We showed that PLP deficiency in both models of diabetes caused severe chromosome and DNA damage, probably through AGE accumulation in turn caused by high glucose levels.

These results allowed to generalize the concept that PLP deficiency combined with high glucose strongly impairs genome integrity, suggesting that in diabetic patients low PLP levels may represent a cancer risk factor.

## Results

### Generation of diabetic fly models

**Figure 1:**
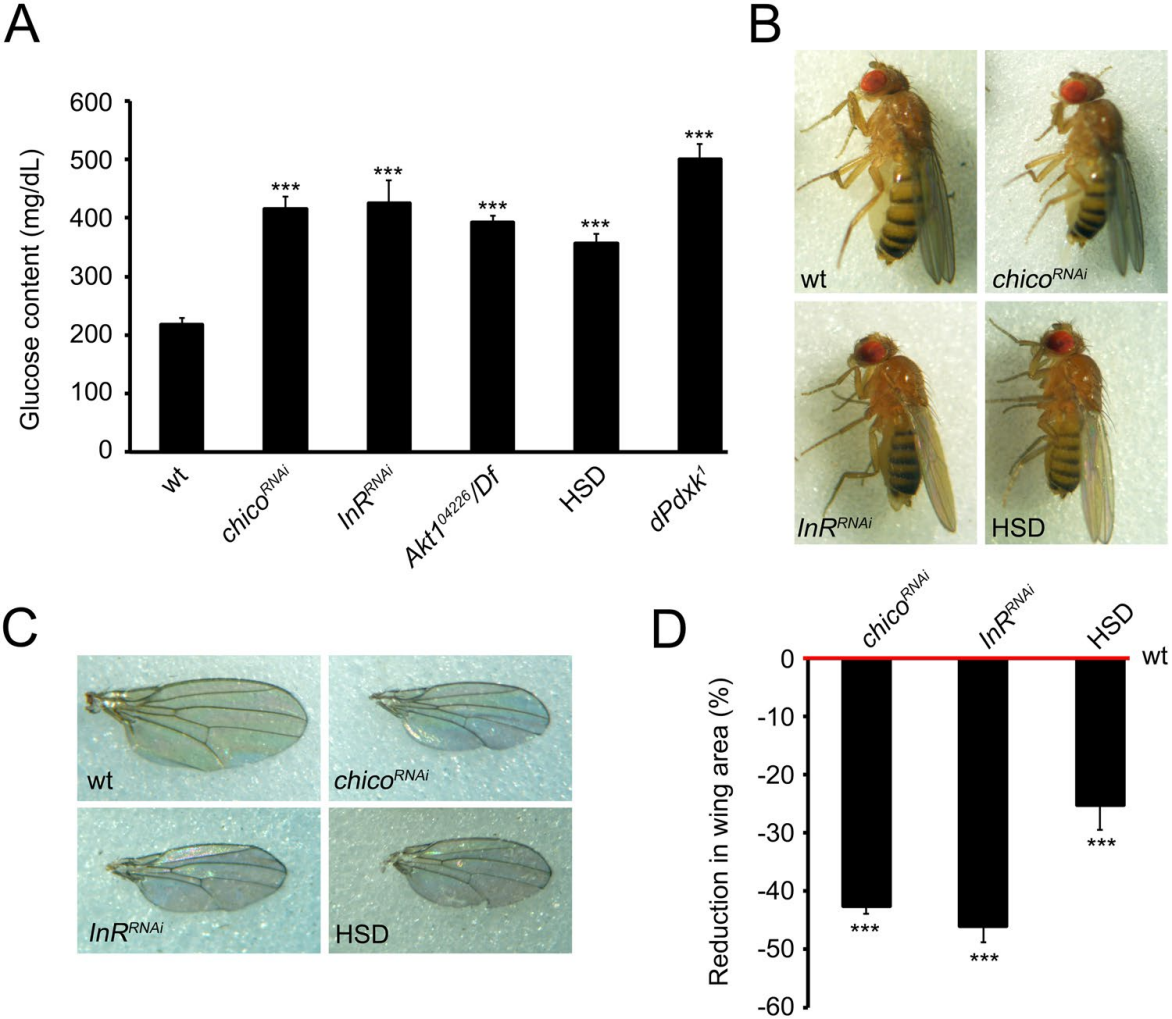
Diabetic hallmarks caused by insulin signaling impairment and high sugar diet (HSD) feeding. (**A**) Glucose content in hemolymph from wild type (wt), RNAi/mutant and HSD fed larvae. *dPdxk*^*1*^ was used as positive control. Columns are the means of 5 independent sample (20 larvae each) measurements ± SEM. ***Significantly different in the Student’s t test with p < 0.001. (**B**) Examples of body size reduction observed in *InR*^*RNAi*^, *chico*^*RNAi*^ and HSD fed flies compared to wt control. (**C**) Examples of wing size reduction observed in *InR*^*RNAi*^, *chico*^*RNAi*^ and HSD fed flies compared to wt control. (**D**) Quantification of wing area reduction. Columns represent mean values of wing area reduction in *InR*^*RNAi*^, *chico*^*RNAi*^ and HSD fed flies compared to wt control. At least 30 wings per genotype were examined ***significantly different in the Student’s t test with p < 0.001.

We previously demonstrated that low PLP levels, due to mutations in the encoding Pyridoxal kinase gene (*dPdxk*), cause CABs and increase glucose content in larval hemolymph^23^. We provided evidence that these phenotypes are linked because high glucose causes AGE accumulation which in turn is responsible for CAB phenotype^23^. Starting from these results, we asked whether low vitamin B6 levels could be genotoxic in diabetes. To answer to this question, we generated two fly models of type 2 diabetes (reviewed in^31,34^) using two strategies. In the first we silenced three evolutionarily conserved genes encoding proteins working in the insulin signaling pathway: Insulin receptor (InR), Insulin receptor substrate (Chico/IRS) and Akt1 (reviewed by^32^). In the second we reared wild type larvae on a high sugar diet (HSD) according to Musselman *et al*.^35^. To deplete InR and Chico we performed *in vivo* RNA interference (RNAi) in fat body (using the *ppl-Gal4* driver), an organ analogue to vertebrate adipose tissue and liver, crucial for nutrient storage and energy metabolism. Targeted gene knockdown was detected by reverse transcriptase-PCR (see Supplementary Fig. S1). The effects of Akt1 depletion were evaluated using the hypomorphic late lethal allele *Akt1*^*04226*^ and all experiments reported in this paper were conducted on hemizygous individuals (*Akt1*^*04226*^*/Df*) to detect stronger phenotypes. Although in flies the most abundant circulating sugar is trehalose (a disaccharide formed by two glucose units) we focused on glucose as trehalose is a stable molecule and a non-reducing sugar^36^. In addition, evidence showed that glucose but not trehalose levels are regulated by mechanisms similar to those of higher organisms^37^. Glucose content in hemolymph from *InR*^*RNAi*^, *chico*^*RNAi*^ *Akt1*^*04226*^*/Df* and HSD fed larvae was increased with respect to wild type and comparable to that exhibited by *dPdxk*^1^ positive control^23^ (Fig. 1A). In addition, InR and Chico RNAi-induced depletion, as well as HSD feeding, resulted in small size adults, a fly hallmark of insulin signaling impairment^38–40^ (Fig. 1B–D; Supplementary Fig. S2). It was not possible to analyze body size in *Akt1*^*04226*^*/Df* individuals as they die as third instar larvae.

### Glucose treatment induces CABs in diabetic cells

**Figure 2:**
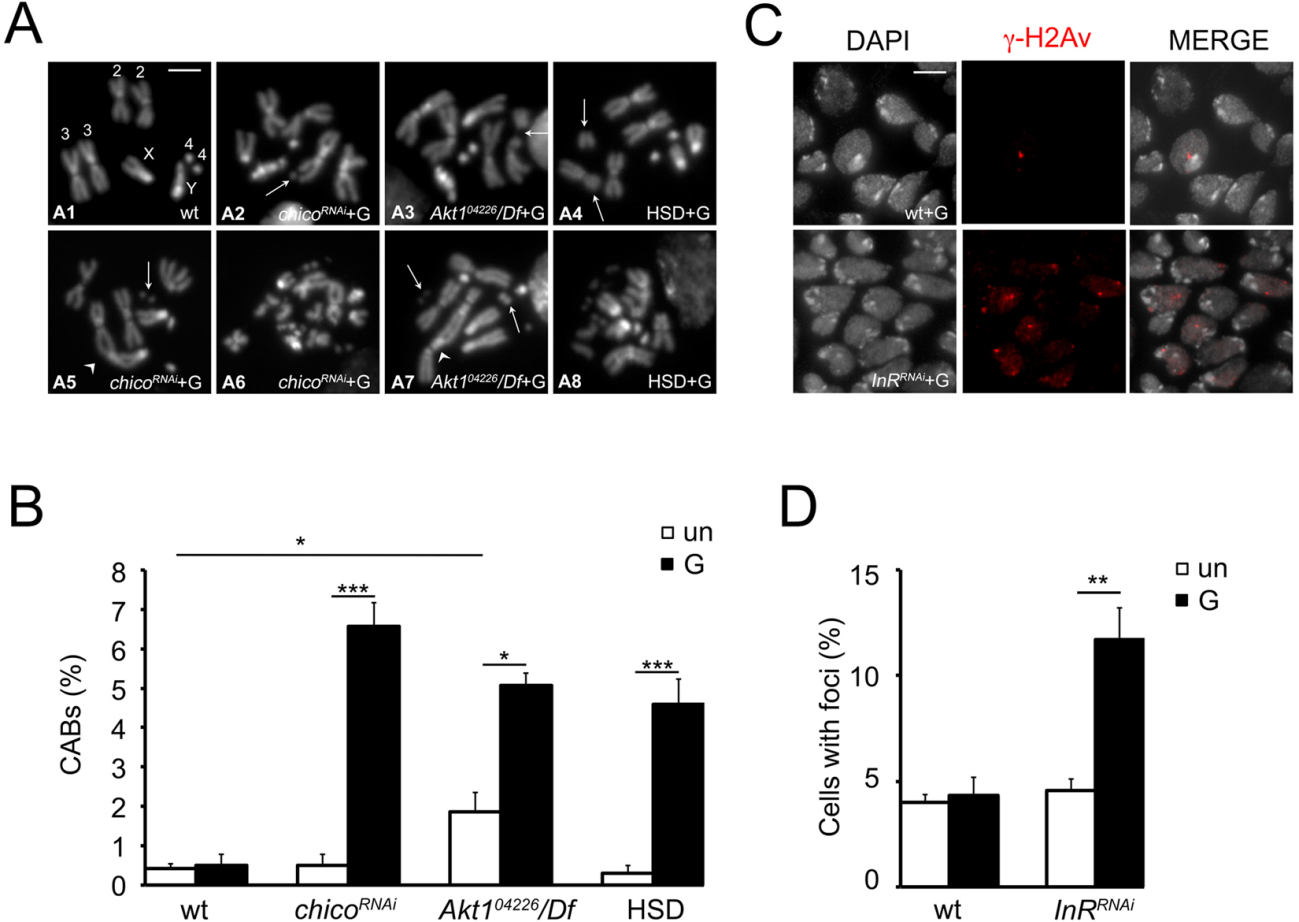
Glucose supplement induces DNA damage in brains from *chico*^*RNAi*^, *Akt1*^*04226*^*/Df*, *InR*^*RNAi*^, and HSD fed larvae. (**A**) Examples of CABs observed in 1% glucose (G) treated brains from *chico*^*RNAi*^, *Akt1*^*04226*^*/Df* and HSD fed larvae. *Df* is *Df(3)Exel7328* that uncovers *Akt1*. (A1) wild type male metaphase; (A2) chromatid deletion of a major autosome, arrow; (A3) isochromatid deletion of a major autosome, arrow; (A4) isochromatid deletion of a major autosome, arrows; (A5) autosome-X chromosome dicentric chromosome (arrowhead) with acentric fragments, arrow; (A6) metaphase with extensive chromosome fragmentation; (A7) autosome-autosome dicentric (arrowhead) chromosome with acentric fragments (arrow) and isochromatid deletion of the X chromosome (arrow); (A8) metaphase with extensive chromosome fragmentation. Scale bar 5 μm. (**B**) CAB frequencies. Un = untreated. G = 1% glucose. Each bar of the graph is the mean frequency of CABs ± SEM obtained examining at least 800 metaphases in at least 8 brains. * and *** significantly different in the Student’s t test with p < 0.05 and p < 0.001 respectively. (**C**) Examples and (**D**) frequencies of γ-H2Av foci observed in wild-type (wt) and *InR*^*RNAi*^ brain nuclei untreated (un) and 1% glucose (G) treated. Bars show the mean values of three independent experiments ± SEM obtained by examining at least 500 cells/brain in 4 brains. **Significantly different in the Student’s t test with p < 0.01.

Evidence shows that glucose induces chromosome damage^41,42^. Thus, we first asked whether the high glucose content present in InR, Chico and Akt depleted individuals and also in HSD fed larvae affected DNA integrity. DNA damage was evaluated in two different ways. In the first we examined chromosomes from *Akt1*^*04226*^*/Df*, *chico*^*RNAi*^ and HSD fed third instar larvae in colchicine treated brain preparations (Fig. 2A), in the second, because of the low mitotic index induced by *InR* silencing, we examined γ-H2Av foci in *InR*^*RNAi*^ brains fixed and immunostained with the pS137 anti-phospho-histone antibody that specifically recognizes γ-H2Av^43^ (Fig. 2C). γ-H2AV is the homolog of the mammalian γ-H2AX and represents a suitable marker to study DNA damage since it marks the DSBs^44^, which are known to be at the basis of CABs^45^.

Neuroblasts from *chico*^*RNAi*^ and HSD fed larvae exhibited frequencies of cells with broken chromosomes comparable to that of controls (0.5% and 0.3% respectively vs 0.44%) (Fig. 2B). *Akt1*^*04226*^*/Df* cells displayed a small but significant CAB frequency (1.86%) (Fig. 2B). In this case CABs are not due to the high endogenous glucose content since *Akt1*^*04226*^*/Df* mutants displayed hemolymph glucose levels comparable to those exhibited by *chico*^*RNAi*^ and HSD fed larvae (Fig. 1A).

Since we previously showed that sugars such as glucose, fructose and sucrose strongly increased CAB frequency in *dPdxk*^1^ neuroblasts^23^, we incubated brains from *Akt1*^*04226*^*/Df*, *chico*^*RNAi*^ and HSD fed larvae in 1% glucose and we tested them for CABs. In all examined cases (Fig. 2A,B) we found CABs ranging from 4.6% to 6.6%; in contrast glucose did not affect chromosome integrity in wild type neuroblasts, indicating that only diabetic cells are sensitive to extra sugar. Glucose treatment generated complex rearrangements; besides chromatid and isochromatid deletions (Fig. 2A2–A4), we observed dicentric chromosomes accompanied by acentric fragments (formed when two broken non homologous chromosomes join together end to end) and also cells containing more than one rearrangement (Fig. 2A5–A8). Glucose induced DNA damage also in InR depleted cells. While *InR*^*RNAi*^ untreated larvae showed a percentage of γ-H2Av positive nuclei (4.5%) comparable to controls (4%), glucose treatment significantly increased this frequency (11.7% *vs* 4.3% in controls) (Fig. 2C,D).

Together these data indicate that the addition of extra glucose to brains from *chico*^*RNAi*^ *Akt1*^*04226*^*/Df*, *InR*^*RNAi*^ and HSD fed larvae causes chromosome damage differently than wild type, suggesting that diabetic cells are sensitive to glucose for CAB formation.

### PLP deficiency induces high levels of DNA damage in diabetic cells

**Figure 3:**
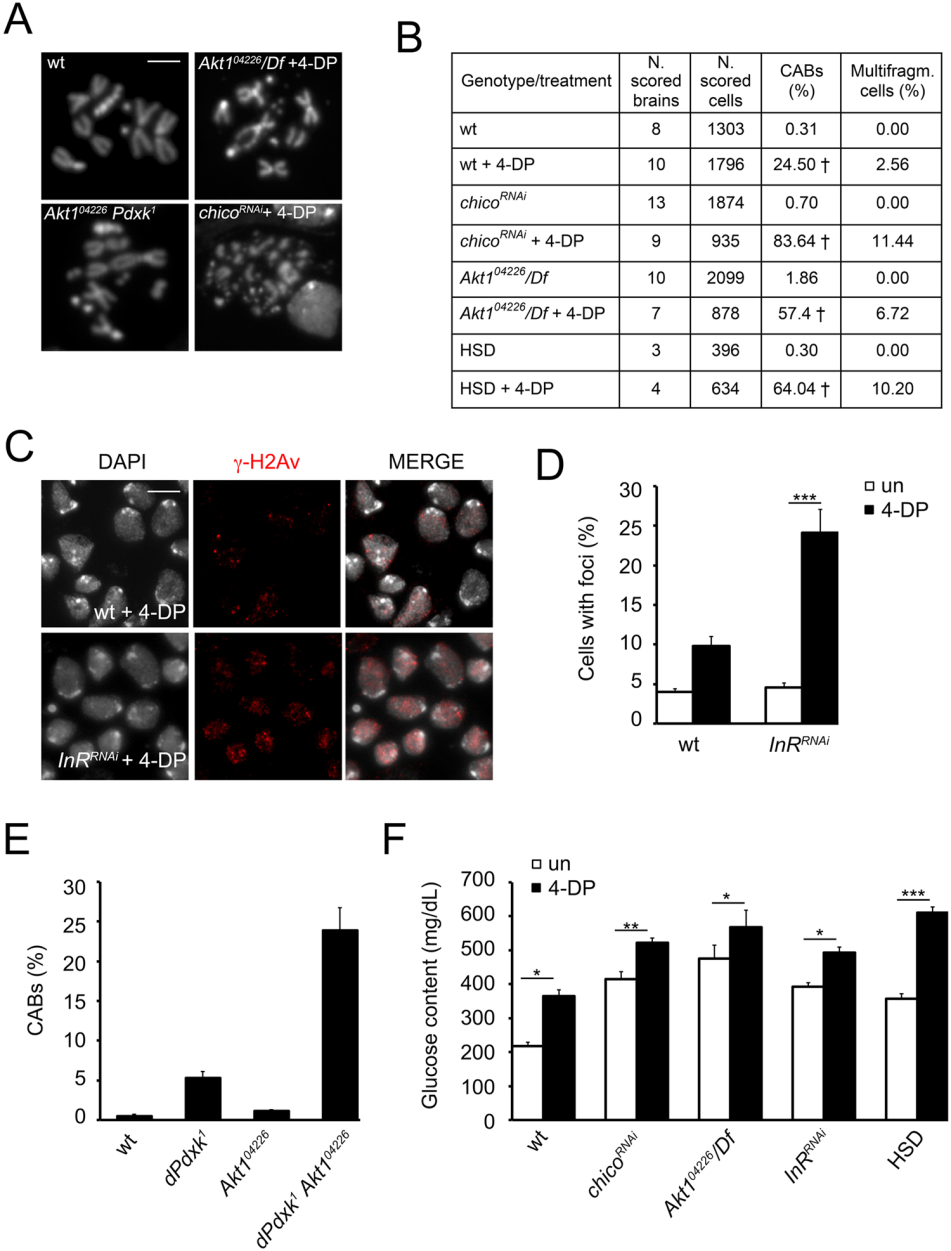
Effect of PLP deficiency on chromosome and DNA integrity in diabetic larvae. (**A**) Examples of complex rearrangements and metaphases with multifragmented chromosomes observed in *chico*^*RNAi*^ and *Akt1*^*04226*^ /*Df* neuroblasts from larvae fed with 4-DP 2 mM and from *Akt1*^*04226*^ *dPdxk*^*1*^ double mutant larvae (Scale bar 5 μm). (**B**) CAB frequencies. ^†^Significantly different in the Student’s t test with p < 0.001 compared to untreated counterparts. (**C**) Examples and (**D**) frequencies of γ-H2Av positive cells from wild type (wt) and *InR*^*RNAi*^ untreated (un) and 4-DP 2 mM fed larvae. Bars show the mean values of three independent experiments ± SEM obtained by examining at least 500 cells/brain in 4 brains. ***Significantly different in the Student’s t test with p < 0.001. (**E**) CAB frequencies in *Akt1*^*04226*^ *dPdxk*^*1*^ double mutant brains. Each column is the mean frequency of CABs ± SEM obtained examining at least 600 metaphases in at least 6 brains. *Akt1*^*04226*^ *dPdxk*^*1*^ CAB frequency was significantly higher than the sum of single mutant CAB frequency in Chi square test with p < 0.01. (**F**) Glucose content in hemolymph from 4-DP treated *InR*^*RNAi*^, *chico*^*RNAi*^, *Akt1*^*04226*^/*Df* and HSD feed larvae. *,** and *** significantly different in the Student’s t test with p < 0.05, p < 0.01 and p < 0.001 respectively.

Our primary aim was to verify whether it is possible to generalize the assumption that a simultaneous occurrence of high glucose and low PLP affects genome integrity. Thus, we examined chromosome and DNA damage in our diabetic models deprived of PLP. To deplete PLP we employed two strategies: in the first we fed *Akt1*^*04226*^*/Df*, *InR*^*RNAi*^, *chico*^*RNAi*^ and HSD reared larvae with a strong PLP antagonist, the 4-Deoxypyridoxine (4-DP), and in the second we generated *Akt1*^*04226*^*dPdxk*^*1*^ double mutant individuals performing genetic crosses. 4-DP caused a substantial increase in chromosome breakage in *chico*^*RNAi*^ (~83-fold) *Akt1*^*04226*^*/Df* (~55-fold) and HSD larvae (~64-fold) (Fig. 3A,B) clearly indicating that PLP deficiency strongly impacted on chromosome integrity in both diabetes models. A significant, although lower, CAB increase (~24-fold) were also found in wild type 4-DP treated cells in agree with the PLP role in genome integrity maintenance, already demonstrated by our and other studies^23,24^. 4-DP enhanced CAB complexity more than glucose and produced cells with extensive chromosome fragmentation (Fig. 3A). As exactly quantifying the number of CABs in these cells was difficult, we decided to arbitrarily assign only five CABs to each cell with multifragmented chromosomes. As a consequence, CAB percentage reported in Fig. 3B may represent an underestimation. 4-DP also enhanced the frequency of γ-H2Av positive cells in *InR*^*RNAi*^ larvae (24 *vs* 9.8% in control) (Fig. 3C,D). Interestingly most of 4-DP treated nuclei presented a foci pattern very similar to X rays-treated cells^46^ with a mean of about 40 foci per cell that is consistent with the complexity of chromosome rearrangements observed in 4-DP treated brains from *chico*^*RNAi*^, *Akt1*^*04226*^*/Df* and HSD fed individuals.

The examination of *Akt1*^*04226*^*dPdxk*^1^ brain cells revealed a CAB frequency (23%) higher than the sum of the frequencies displayed by each single mutant (*Akt1*^*04226*^ 1.1%; *dPdxk*^*1*^ 5%) (Fig. 3E). These data suggest a strong synergistic interaction between *dPdxk*^*1*^ and *Akt1*^*04226*^ mutations confirming the hypothesis that CABs originate by a combination of high glucose and low vitamin B6 levels accordingly with results obtained with 4-DP.

Based on the consideration that *dPdxk*^*1*^ mutants contain more glucose than wild type strain we asked if PLP depletion could further increase glucose level in larval hemolymph from *Akt1*^*04226*^*/Df*, *InR*^*RNAi*^, *chico*^*RNAi*^ and HSD fed individuals. As showed in Fig. 3F, 4-DP fed larvae exhibited a small but significant glucose content increase, with respect to untreated individuals.

Taken together these results indicate that in diabetic cells PLP deficiency strongly impairs DNA integrity.

### AGEs contribute to DNA damage occurring in diabetic cells deprived of PLP

**Figure 4:**
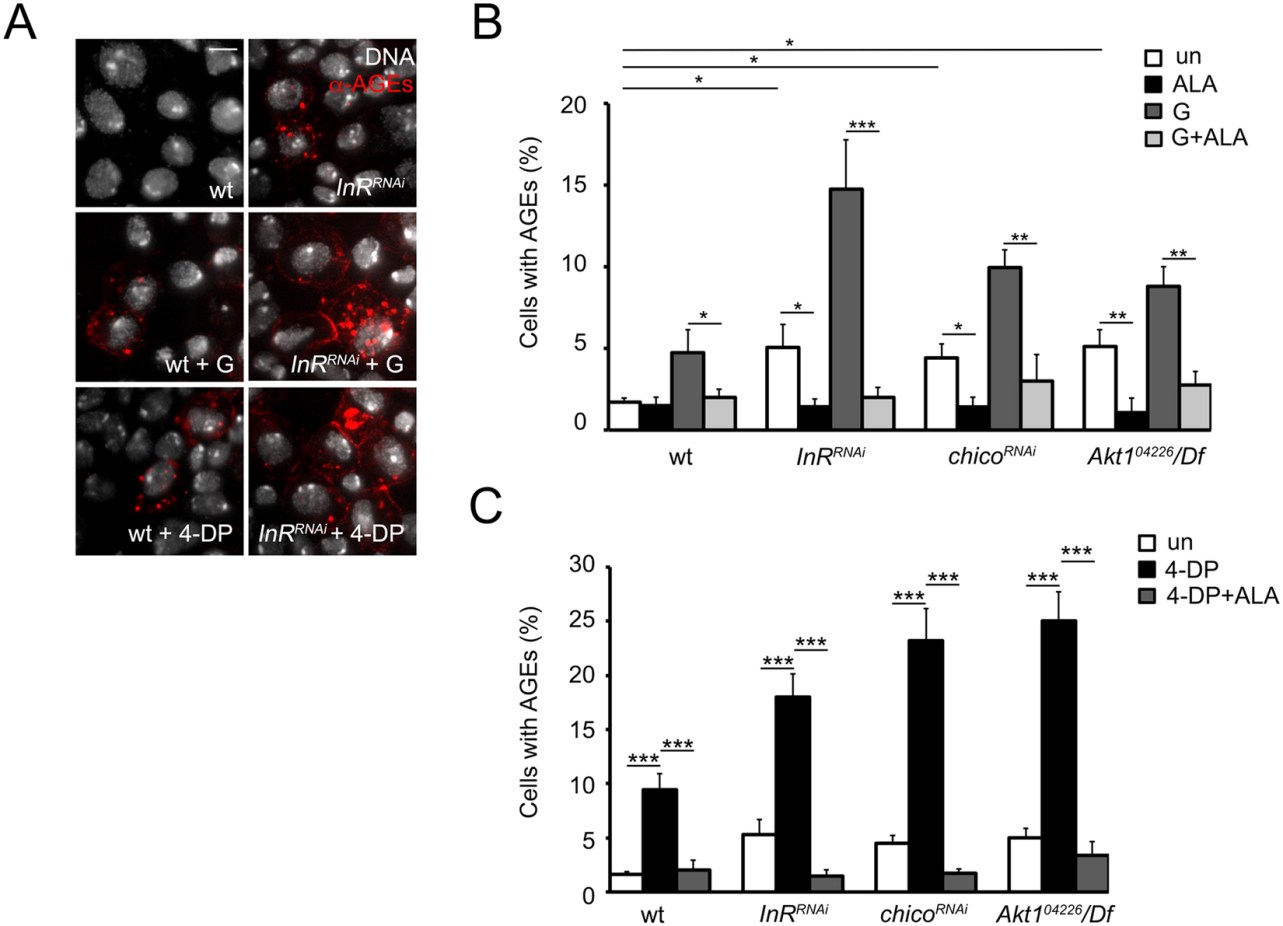
*InR*^*RNAi*^, *chico*^*RNAi*^ and *Akt1*^*04226*^/*Df* larvae accumulate AGEs that are increased by glucose and 4-DP treatment. (**A**) Examples of cells stained with an anti-human AGE antibody from untreated (un), 1% glucose (G) treated and 4-DP fed larvae. Scale bar, 5 μm. (**B**) Frequencies of AGE-positive cells in wild type (wt), *InR*^*RNAi*^, *chico*^*RNAi*^ and *Akt1*^*04226*^/*Df* mutant brains untreated and exposed to 1% glucose (G) with or without α-lipoid acid (ALA). ALA treatment reduces drastically AGEs. (**C**) Frequencies of AGE-positive cells in wild type (wt), *InR*^*RNAi*^, *chico*^*RNAi*^ and *Akt1*^*04226*^/Df mutant brains from 4-DP fed larvae untreated (un) and treated with ALA. Bars in B and C graphs represent the mean frequencies of AGE-positive cells (±SEM) obtained by examining at least 300 cells/brain in 4 brains. *,** and *** significantly different in the Student’s t test with p < 0.05, p < 0.01, p < 0.001 respectively.

**Figure 5:**
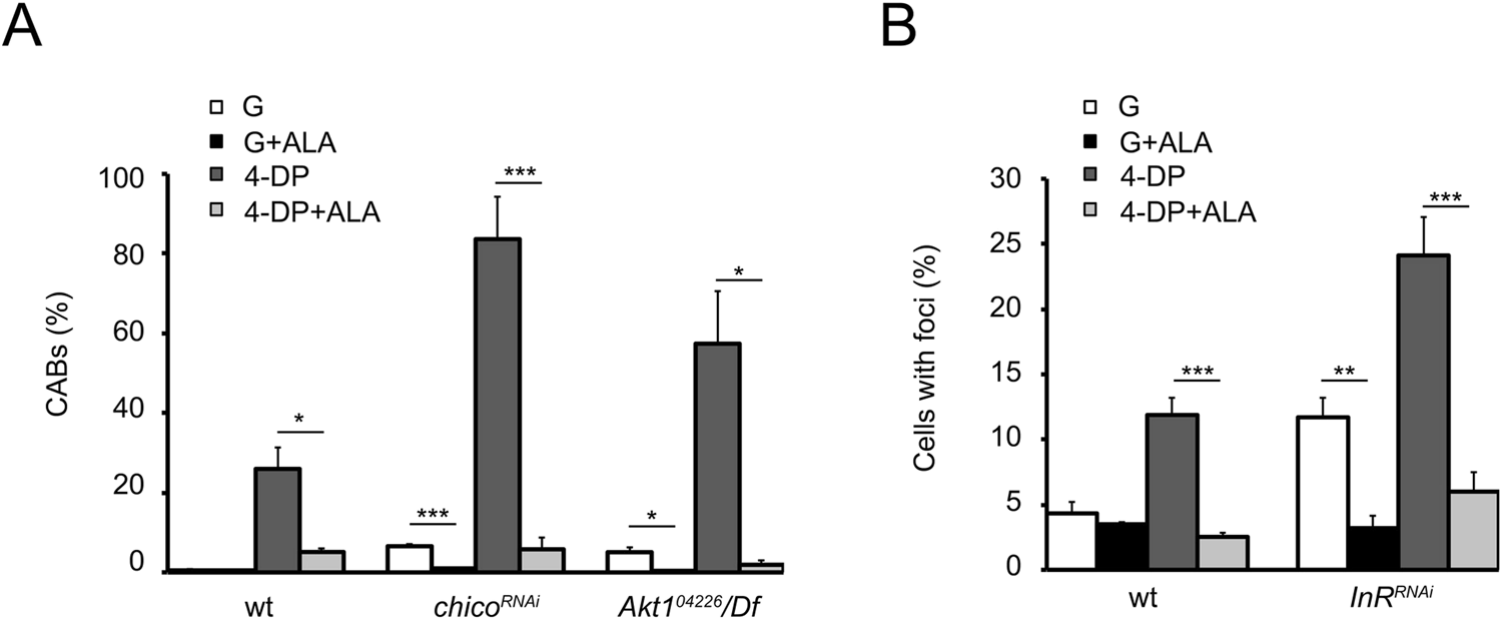
α-lipoid acid (ALA) reduces DNA damage in *InR*^*RNAi*^, *chico*^*RNAi*^ and *Akt1*^*04226*^*/Df* neuroblasts. (**A**) Effect of ALA on CAB frequencies in wild type (wt) *chico*^*RNAi*^ and *Akt1*^*04226*^/*Df* neuroblasts 1% glucose (G) and 4-DP treated. Columns represent the mean frequency of CABs (±SEM) obtained by examining at least 600 metaphases from at least 6 brains. * and *** significantly different in the Student’s t test with p < 0.05 and p < 0.001. (**B**) Effect of ALA treatment on γ-H2Av foci in wt and *InR*^*RNA*i^ neuroblasts 1% glucose (G) and 4-DP treated. Bars show the mean values of three independent experiments ± SEM. ** and *** significantly different in the Student’s t test with p < 0.01 and p < 0.001.

It is well known that PLP counteracts the accumulation of AGEs, genotoxic compounds abundant in most cells and fluid of diabetic patients^14,15,47^. AGEs originate from non-enzymatic amino group glycations of proteins, lipids and nucleic acids^48,49^, that accumulate during senescence and in high glucose conditions and their formation have been associated to reactive oxygen species production^26^. To investigate the mechanism through which PLP protects from damage in high glucose conditions we immunostained untreated, 1% glucose and 4-DP treated brains from *InR*^*RNAi*^, *Chico*^*RNAi*^ and *Akt1*^*04226*^*/Df* larvae using a human antibody against AGEs. We did not test HSD larvae for AGEs since we expected that there is the same mechanism at the basis of CAB formation in both models of diabetes. InR, Chico or Akt1 depleted neuroblasts showed AGE accumulation (Fig. 4A,B) more pronounced after glucose treatment. 4-DP feeding further increased the frequency of cells positive to the anti-AGE staining in all examined cases (Fig. 4A,C). We next incubated *Akt1*^*04226*^*/Df*, *InR*^*RNA*i^ and *chico*^*RNAi*^ brains in 10 mM α-lipoic acid (ALA), an antioxidant compound that counteracts AGE formation and cooperates with PLP in ameliorating insulin resistance in prediabetic rats^50,51^. In all cases ALA treatment decreased AGE accumulation in both glucose and 4-DP treated cells (Fig. 4B,C). More interestingly ALA also decreased DNA damage. As showed in Fig. 5A, in *Akt1*^*04226*^*/Df*, and *chico*^*RNAi*^cells ALA treatment reduced CAB frequency to control values in both glucose treated and 4-DP fed larvae. Similarly, ALA also lowered the percentage of *InR*^*RNA*i^ γ-H2Av positive cells in neuroblasts from glucose treated and 4-DP fed larvae (Fig. 5B). These results suggested a cause effect relationship between AGEs and CABs and indicated that AGEs are largely responsible for chromosome and DNA damage occurring in diabetic PLP depleted cells.

### PLP rescues glucose-induced DNA damage in diabetic cells

**Figure 6:**
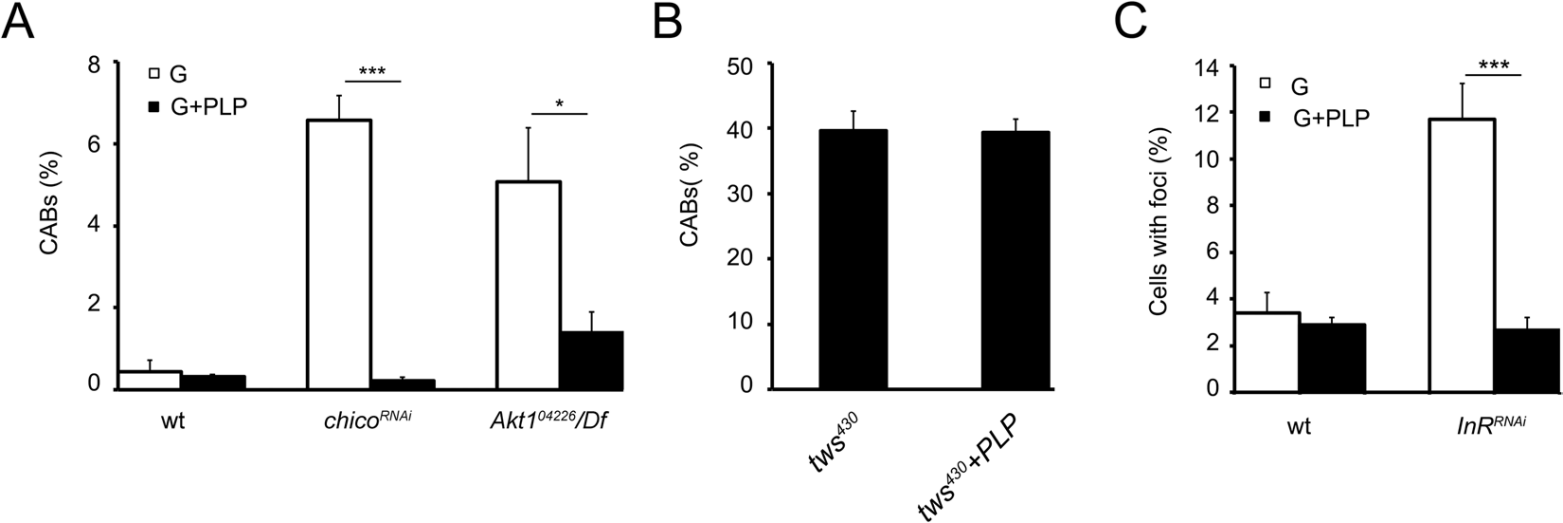
PLP rescues CABs and γ-H2Av foci in *chico*^*RNAi*^, *Akt1*^*04226*^*/Df* and *InR*^*RNAi*^ brains. (**A**) Effect of PLP on CAB frequencies in 1% glucose (G) treated wild type (wt), *chico*^*RNAi*^ and *Akt1*^*04226*^/*Df* larval neuroblasts. Columns represent the mean frequency of CABs (±SEM) obtained by examining at least 600 metaphases from at least 6 brains. * and *** significantly different in the Student’s t test with p < 0.05 and p < 0.001 respectively. (**B**) Effect of PLP on *tws*^430^ CAB frequency. Columns represent the mean frequency of CABs (±SEM) obtained by examining at least 500 metaphases from at least 5 brains. (**C**) Effect of PLP on γ-H2Av foci in wt and *InR*^*RNAi*^ 1% glucose (G) treated neuroblasts. Bars show the mean values of three independent experiments ± SEM. *** significantly different in the Student’s t test with p < 0.001.

We previously demonstrated that PLP supplementation in both untreated and glucose treated *dPdxk*^*1*^ mutants rescued AGE accumulation confirming the role of vitamin B6 in counteracting these compounds also in *Drosophila*^23^. Based on these findings, we asked if PLP supplementation could reduce chromosome and DNA damage in *Akt1*^*04226*^*/Df*, *chico*^*RNAi*^ and *InR*^*RNAi*^ glucose treated brains. Thus, we reared mutant/RNAi larvae in food supplemented with 10^−2^ M PLP and examined them for CABs or γ-H2Av foci. To ascertain the PLP specificity we also tested brains from PLP fed individuals bearing *twins* (*tws*) mutation that causes a high frequency of CABs (about 40%) impairing a pathway uncorrelated with glucose metabolism^46^. PLP rescued glucose induced chromosome breakage in *Akt1*^*04226*^*/Df* and *chico*^*RNAi*^ cells (Fig. 6A) but it did not influence CAB frequency in t*ws*^*430*^ mutant brains (Fig. 6B) confirming its specificity. PLP also rescued the percentage of *InR*^*RNAi*^ glucose treated γ-H2Av positive cells (Fig. 6C).

Taken together these data indicate that vitamin B6 improves glucose-induced DNA damage and strongly support the hypothesis that PLP is involved in chromosome integrity maintenance in high glucose conditions.

**Figure 7:**
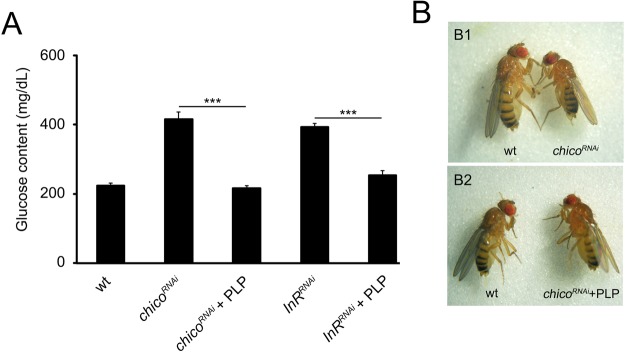
PLP rescues glucose content and small body size in diabetic individuals. (**A**) Glucose content in *InR*^*RNAi*^ and *chico*^*RNAi*^ larval hemolymph after PLP treatment. Columns are the means of 5 independent sample (20 larvae each) measurements ± SEM. *** significantly different in the Student’s t test with p < 0.001. (**B**) PLP treatment rescues body size in *chico*^*RNAi*^ and *InR*^*RNAi*^ individuals. B1 Example of small body size in *chico*^*RNAi*^ (or *InR*^*RNAi*^); B2 rescued body size in *chico*^*RNAi*^ (or *InR*^*RNAi*^) flies treated with PLP.

Remarkably PLP also rescued glucose content and body size in *InR*^*RNAi*^ and *Chico*^*RNAi*^ individuals suggesting that vitamin B6 plays a role in ameliorating the tissue sensitivity to insulin (Fig. 7A,B). These findings are consistent with our previous data^23^ indicating that *Pdxk*^*1*^ mutations cause a mild insulin resistance. Mechanisms linking PLP to insulin resistance will be the subject of future studies.

## Discussion

For the past decade, *Drosophila* has been fast gaining popularity as a useful model system to study diabetes as it shares with humans mechanisms and molecules involved in hemolymph/blood glucose regulation. In *Drosophila* stable levels of circulating sugars are maintained by eight insulin-like proteins (Ilps) that are released in response to high glucose levels and by the adipokinetic hormone (AKH) that it is similar to glucagon and is released in response to low levels of circulating sugar^52–55^. Type 1 and 2 diabetes have been modeled in *Drosophila* reproducing hallmarks of both diseases^35,56–58^ giving the chance to isolate new genes and identify functional interactions that can be exploited for therapeutic interventions on human patients.

We demonstrated that PLP deficiency due to mutations in *dPdxk Drosophila* gene causes DNA damage and increases glucose content in larval hemolymph. We showed that these phenotypes are correlated and that high glucose is largely responsible for DNA damage. In addition we showed that also in human HeLa cells the relationship between PDXK, DNA integrity and glucose content has been maintained in the course of evolution^23^.

In the present work we have provided evidences that PLP deficiency in fly diabetic cells strongly impair DNA integrity. We demonstrated the combined genotoxic effect of low PLP and high glucose levels using two different diabetes models, the first obtained by downregulating the expression of three well conserved insulin pathway genes: *InR*, *chico* and *Akt1*, and the second obtained by feeding flies with a high glucose diet (HSD). Both insulin signal impairment and HSD feeding are considered promising strategies to model type 2 diabetes in flies^34^. Glucose levels obtained by depleting InR, Chico or Akt1 were similar to those obtained by Ugrankar *et al*.^37^, although we used a different fat body driver to silence *InR* and *chico* and also a different *Akt*1 allele.

We showed that high level of endogenous glucose caused by either insulin signaling impairment or HSD feeding is not sufficient to compromise DNA integrity. A possible explanation of this can be that fly metabolism relies mainly on treahalose that represents the more abundant circulating not-reducing sugar with ratio 100:1 with respect to glucose. Thus, to observe glycation effects the system need to be forced by adding more glucose. We showed in fact that glucose treatment in Inr, Chico or Akt1 depleted brains as well as in neuroblast from HSD fed larvae caused CABs or γ-H2Av foci. In contrast glucose did not influence chromosome integrity in wild type cells suggesting that diabetic cells are more sensitive to glucose respect to wild type cells. We found that *Akt1*^*04226*^*/Df* mutants displayed a low frequency of CABs also in untreated cells. However, based on evidence indicating that the human AKT counterpart is involved in DNA integrity maintenance^59^ we think that CABs in these cells are not related to glucose metabolism, but are due to an improper DNA repair.

More importantly, we demonstrated that in both our hyperglycemic models, DNA damage was exacerbated when PLP levels were decreased by 4-DP, a strong PLP antagonist. 4-DP caused a dramatic CAB increase in *chico*^*RNAi*^ (83.6%), *Akt1*^*04226*^*/Df* (57.4%) and HSD larval brains (64%) and also enhanced about 5 times the percentage of γ-H2AV positive cells in *InR*^*RNAi*^ individuals with respect to untreated counterparts. 4-DP further increased glucose content that, combined with an impaired antioxidant defense system, caused by PLP deficiency, may contribute to amplify chromosome and DNA damage. In addition we provided genetic evidence of a strong synergistic interaction between *Akt1* and *dPdxk*^*1*^ mutations in CAB formation in double mutant individuals, accordingly with results obtained using 4-DP.

These results demonstrate that it is possible to extend the concept that low PLP levels cause DNA damage in high glucose conditions also to other diabetic contexts different from those caused by *Pdxk*^*1*^ mutation^23^.

Regarding the mechanism leading to CABs in diabetic PLP deprived cells we identified the pathway that leads to AGE formation as the best candidate that allow to correlate PLP, glucose and DNA damage. We showed that *InR*^*RNAi*^, *chico*^*RNAi*^ or *Akt1*^*04226*^*/Df* diabetic cells accumulate AGEs which in turn are reduced by α-lipoic acid (ALA). More interestingly ALA also rescued both CABs and γ-H2Av foci suggesting a cause effect relationship between AGEs and CABs. As AGEs are known to be genotoxic through oxygen reactive species formation, these findings indicate that in diabetic cells PLP may safeguard genome integrity preventing AGEs from attack DNA. Our hypothesis is based on *in vitro*^15^ and *in vivo* studies showing that PLP is able to counteract AGE formation. It has been reported that PLP inhibits AGE accumulation in streptozotocin-induced diabetic rats, preventing the progression of nephropathy^60^. In addition, in glomerular cell nuclei of these rats PLP reduced the levels of carboxyethyl-2′deoxyguanosine (CEdG), a DNA glycation product that can cause strand breaks^61^. The exact mechanism through which PLP counteracts AGEs is not to date fully elucidated. A hypothesis is that PLP may block AGE formation by trapping the 3 deoxyglucosone (3-DG), an intermediate product of AGE metabolism. It has been in fact indicated, by *in vitro* experiments, that PLP is able to sequestrate 3-DG in a dose dependent manner^62^. However also other mechanisms are possible. PLP also works as a cofactor of enzymes that convert dUMP in dTMP. *dPdxk*^*1*^ mutants have an unbalanced amount of dUMP respect to dTMP^23^ but as are not sensitive to replication block induced by HU, this excess of dUMP does not seem to be the principal mechanism that leads to CABs. Anyway, we cannot exclude that nucleotide unbalance caused by PLP depletion can slightly affect chromosome stability in our diabetes models by influencing the rate of DNA repair. Furthermore, to the hypersensitivity of diabetic cells to 4-DP toxicity can contribute an impairment of DNA repair associated to diabetes. It has been demonstrated that diabetic patients showed, besides high levels of basal endogenous and oxidative damage, an increased sensitivity to DNA damaging agents combined to a decreased capability to repair damage induced by these drugs^63^.

Altogether our data, extrapolated to human, strongly support the hypothesis that DNA damage represents one of the causative factors linking diabetes to cancer. In addition, they highlight the crucial role of PLP in chromosome stability maintenance in diabetic cells suggesting that low PLP levels can represent a critical cancer risk factor for diabetic patients. Low PLP levels in normal physiology represent a rare condition due to excessive consumption of alcohol, drugs commonly used to treat pathologies as tuberculosis or arthritis (e.i. isonyazide, cycloserine, penicillamine) or celiac disease and renal dialysis^64^. However, interestingly, low PLP levels have been associated to diabetes in murine models and epidemiological studies^65–67^. In particular, a recent study^68^ based on the examination of three groups of individuals, diabetic, diabetic with nephropathy and healthy subjects, showed that respectively 63% and 58% of diabetic patients with and without nephropathy presented low PLP plasma levels (<30 nM/L). Moreover, altered levels of PLP precursors pyridoxine and pyridoxamine have been found in diabetic patients suggesting an impaired PLP metabolism associated to diabetes. At the light of these considerations our findings suggest that in diabetic individuals PLP levels should be kept under control in order to limit the risk of DNA damage.

## Methods

### *Drosophila* strains and crosses

*InR*^*v992*^ and *chico*^*v7776*^ lines were obtained from the Vienna *Drosophila* Resource Center (VDRC) stock center. *dPdxk*^*1*^ mutation has been described previously^23^. *ppl-Gal-4* driver^52^, *Akt1*^*04226*^ and *Df Exel 7328* (89A12-89B6) lines were obtained from the Bloominghton Stock Center. *InR*^*RNAi*^ and *chico*^*RNAi*^ flies were generated by crossing females bearing the RNAi construct to males carrying the fat body specific *ppl-Gal4* driver. Mutations and deficiencies were kept in stock over the *TM6C Sb*, *Tb* balancer; homozygous and hemizygous mutant larvae were recognized for their *non-Tubby* phenotype. The *Oregon-R* strain was used as wild-type control.

Double mutants *dPdxk*^1^ *Akt1*^*04226*^ were generated by recombination by crossing *Akt1*^*04226*^*/TM6C* females to *dPdxk*^*1*^*/TM6C* males. *Akt1*^*04226*^/*dPdxk*^*1*^ females resulting from this cross were mated to *Ap*^*Xa*^*/TM6C* males. Single males resulting from this cross were mated to *Ap*^*Xa*^*/TM6C* females. From these crosses were established about 60 stocks crossing inter se males and females carrying recombinant chromosomes balanced over *TM6 C*. The presence of both mutations on the recombinant chromosomes was verified by complementation analysis. The balancers and the genetic markers used in these crosses are described in detail in FlyBase (http://flybase.bio.indiana.edu/).

### Fly food recipes

All flies, from embryo stage, were raised on a standard food. To induce insulin resistance wild type larvae were reared on a sugar rich medium which is a standard food with an increased sucrose concentration. 100 ml food contains: agar (0.68 g), yeast (6.52 g), flour (3 g) propionic acid (600 µl) and sucrose (5.13 g = 0.15 M for standard food; 34.2 g = 1.0 M for high sugar diet, HSD).

### Chromosome cytology

Colchicine-treated larval brain metaphases for CAB scoring were obtained as previously described^69^. For immunostaining, brains from third instar larvae were dissected and fixed as described in Bonaccorsi *et al*.^70^. Brain preparations were rinsed in PBS 0.1% Triton (PBST), incubated overnight at 4 °C with primary antibodies diluted in PBST, rinsed in PBST, and then incubated for 1 hr at room temperature with the secondary antibody. The primary antibodies used were: rabbit anti-Histone H2AvD pS137 (1:100 in PBST; Rockland code #600-401-914) and rabbit anti-human AGE antibody (1:200 in PBST; ab23722, Abcam, UK). Both antibodies were detected with Alexa-Fluor-555-conjugated anti-rabbit antibody (1:300 in PBST; Molecular Probes, Eugene, OR). All fixed preparations were mounted in Vectashield H-1200 with DAPI (Vector Laboratories, Burlingame, CA) to stain the DNA. Observations were carried out using a Zeiss Axioplan fluorescence microscope equipped with CCD camera (Photometrics CoolSnap HQ).

### Drug treatments

To evaluate the effects of PLP inhibitor 4-deoxypyridoxine (4-DP) on CABs and γ-H2Av foci, flies were allowed laying eggs on a fly standard growth medium for 5 days. Then, adults were removed and 4-DP 2 mM was added to medium (containing first and second instar larvae). 5 days later third instar larvae were dissected and brains fixed. For chromosome preparations brains were incubated 90 min in colchicine before fixing. To detect γ-H2Av foci brains were treated according to immunostaining procedure.

To test the effects of glucose, PLP, and α-lipoic acid (ALA) on CABs and γ-H2Av foci, brains were dissected from third instar larvae and incubated in 2 ml of saline supplemented with 10% fetal bovine serum (FBS, Corning) for 4 hours with or without addition of 1% glucose, 1 mM PLP or 10 mM ALA. For chromosome preparations 1 h before fixation brains were treated with colchicine 10^−2^ M (final concentration). For γ-H2Av foci detection brains were treated according to immunostaining procedure.

### Picture acquisition

Pictures of adult flies were taken using a Nikon D5200 digital camera mounted on a stereomicroscope (Nikon SMZ-1). Pictures were taken using a 1/6 second exposure, and 800 iso.

### Glucose and wing measurement

Glucose concentration in *Drosophila* hemolymph was measured using the Infinity Glucose Hexokinase reagent (Thermo scientific). Hemolymph collection and glucose measurement were done as described in Marzio *et al*.^23^.

For wing analysis, flies were anesthetized with CO_2_. Dissected wings were placed in absolute ethanol, and mounted in a 6:5 mixture of lactic acid/ethanol^71^. Measurements were made directly on digitized images of mounted wings using ImageJ software.

### Weight analysis

Body weight of individual male and female flies (n = 20) was measured with a precision weight scale (Gibertini E42; range 0.1 mg-120 g). Flies were reared under the same growth conditions and were age matched (2 days old) before weighing.

### Nucleic acid extraction, PCR, and RT- PCR

Preparation of fly RNA, PCR, RT-PCR and agarose gel electrophoresis were performed with standard procedures. RNA extraction was performed with the RNeasy Mini Kit (Qiagen, Hilden, Germany). For RT-PCR we used 1 μg of RNA to synthesize complementary DNAs (cDNAs) using the Quantitect Reverse transcription kit (Quiagen cod # 205311) For cDNA amplification were used the following primers:

*chico*    F    CTGACATTCGTGTGCATTGGA

*chico*    R    ATGCTTGTTGGTTGAGTGCGG

*InR*    F    GTGCTCCTCCGGTCTTATCGA

*InR*    R    GTGACGTTCAGCATAGCGGAG

*rp49*    F    TACAGGCCCAAGATCGTGAA

*rp49*    R    ACGTTGTGCACCAGGAACTT

### Statistical analysis

Results are expressed as means ± SEM; probability values < 0.05 were considered statistically significant. Statistical analysis of the data was done with the two-tailed Student’s t-test.

### Data availability

The datasets generated during and/or analyzed during the current study are available from the corresponding author on reasonable request.

## Electronic supplementary material

supplementary information
