# Supplementary material for: Protective role of vitamin B6 (PLP) against DNA damage in Drosophila models of type 2 diabetes
Source: Sci Rep. 2018 Jul 30;8:11432. doi: 10.1038/s41598-018-29801-z (PMC6065437; doi:10.1038/s41598-018-29801-z)
Supplement: Supplementary file 1 — supplementary information [file 41598_2018_29801_MOESM1_ESM.pdf]

Supplementary information

Protective role of vitamin B6 (PLP) against DNA damage in *Drosophila* models of type 2 diabetes

Chiara Merigliano[1], Elisa Mascolo[1], Mattia La Torre[1], Isabella Saggio[1,2,&] and Fiammetta Vernì[1,*]

[1] Dipartimento di Biologia e Biotecnologie "C. Darwin" Sapienza Università di Roma, 00185 Italy

[2] Istituto di Biologia e Patologia Molecolari del CNR, Rome 00185 Italy

[&] Co-corresponding author

[*] Corresponding author

[*, &] Correspondence:

Fiammetta Vernì <fiammetta.verni@uniroma1.it>

Isabella Saggio <isabella.saggio@uniroma1.it>

Dipartimento di Biologia e Biotecnologie,

Sapienza, Università di Roma

00185 Roma, Italy

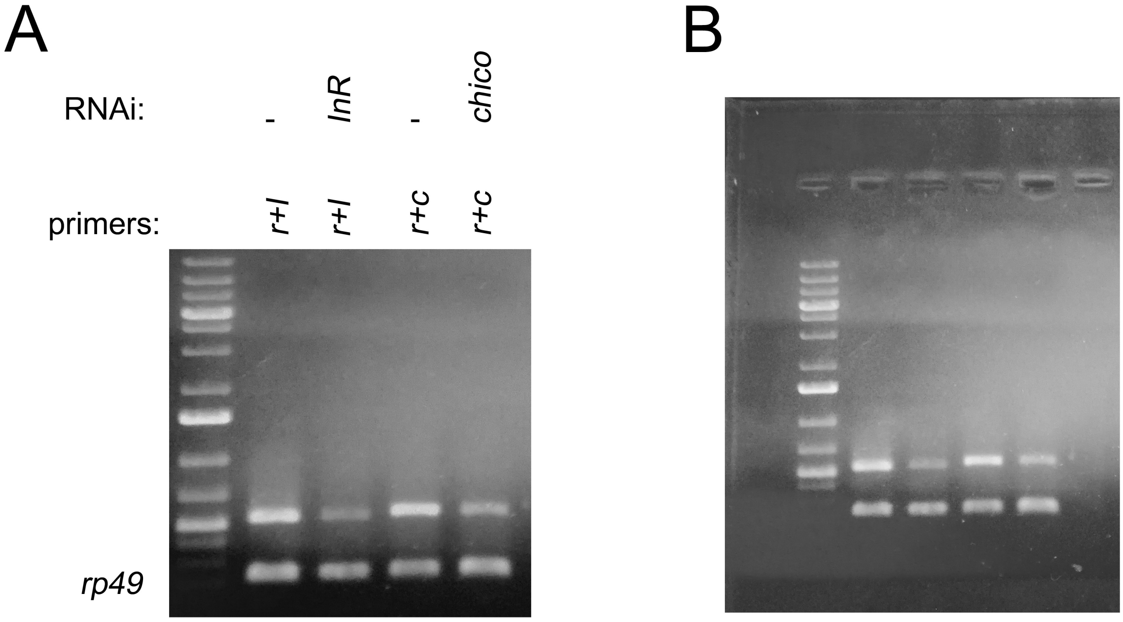

Supplementary Figure S1. RNAi efficiency (A) RT-PCR showing that RNAi against the indicated genes disrupts the corresponding mRNAs. The rp49 mRNA, encoding the Ribosomal protein 49 served as an internal control. The primers used are reported in Materials and Methods and abbreviated as follows: *r, rp49; I*, *InR*; *c*, *chico.* Amplification products are: 558 bp with *chico* primers; 540 bp with *InR* primers; 224 bp with *rp49* primers. The most fluorescent bands of the marker represent from the top to the bottom the molecular weights of: 5000, 1500, 500 bp. **(B)** Original image of the RT-PCR gel.

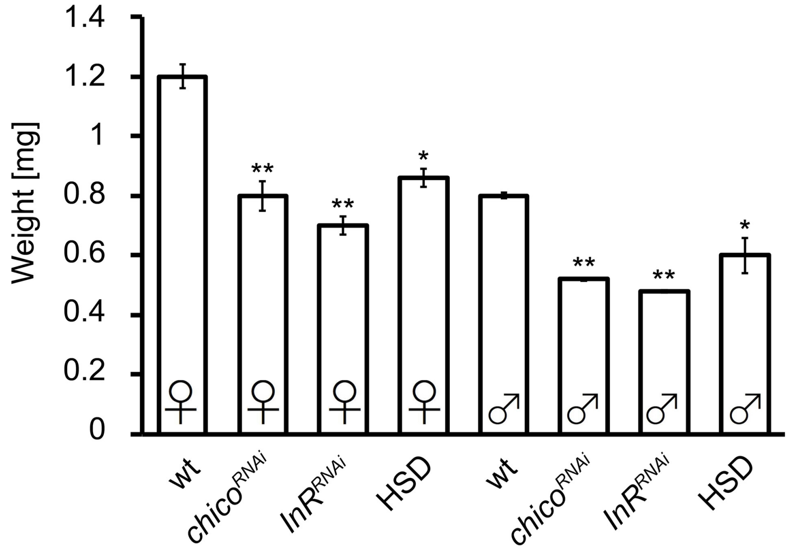

Supplementary Figure S2. Body size reduction Each column represents the mean of the body weight (± SEM) measured in 20 individuals for each genotype. * and ** significantly different in the Student's t test with $p<0.05$ and $p<0.01$.
